# Supplementary material for: The Epidemiology of Mobility Difficulty in Saudi Arabia: National Estimates, Severity Levels, and Sociodemographic Differentials
Source: Healthcare (Basel). 2025 Jul 25;13(15):1804. doi: 10.3390/healthcare13151804 (PMC12346842; doi:10.3390/healthcare13151804)
Supplement: Supplementary file 1 [file healthcare-13-01804-s001.zip › healthcare-3757322 supplementary file 1.pdf]

The Washington Group
Extended Set on Functioning (WG-ES)

Introduction

The Washington Group Extended Set on Functioning (WG-ES) was developed, tested and adopted by the Washington Group on Disability Statistics (WG). The questions reflect advances in the conceptualization of disability and use the World Health Organization's International Classification of Functioning, Disability, and Health (ICF) as a conceptual framework.

The WG-ES is intended for use in population-based health surveys, as well as surveys that focus specifically on disability. It may also be included in surveys that focus on other topics where the survey design is such that:

a) extensive information is collected on selected adult family members; and
b) information is collected directly from the respondent, rather than a proxy, unless the respondent is unable to participate due to a health problem or functional limitation.

To maximize international comparability, the WG-ES obtains information on difficulties a person may have in undertaking basic functioning activities, including seeing, hearing, walking or climbing stairs, remembering or concentrating, self-care, communication (expressive and receptive), upper body activities, affect (depression and anxiety), pain, and fatigue. The WG-ES is comprised of 34 questions, plus an additional 3 'optional' questions, in these ten domains of functioning. The six WG Short Set on Functioning questions are embedded in the WG-ES.

The questions are designed to collect information on the adult population aged 18 years and above. The WG-ES was not designed for use among children, as it does not include key aspects of child development important for identifying disability in children and the wording of certain domains may not be relevant (or suitable) for children and adolescents. The WG-UNICEF Module on Child Functioning was designed specifically to meet the needs of identifying and measuring disability in children.

The Washington Group website [http://www.washingtongroup-disability.com/] contains supporting documentation, including information for translation, cognitive testing, question specifications and interview guidance, and analytic guidelines, including SPSS, SAS and STATA syntaxes.

It is important to note that each question has four response categories, which are to be read after each question.

Preamble to the WG-ES:

Note: The purpose of the introduction is to serve as a transition from questions in the census or survey instrument that deal with other subject matters to this new area of inquiry, and to focus the respondent on difficulties they may have doing basic activities.

Use of the introductory statement may not be needed in all situations, especially if including the statement may interrupt the flow of question administration.

Interviewer read: "The next questions ask about difficulties you may have doing certain activities."

VISION

VIS_1 [Do/Does] [you/he/she] wear glasses?

1. Yes
2. No
7. Refused
9. Don't know

VIS_2 [Do/Does] [you/he/she] have difficulty seeing, [*If VIS_1 = 1:* even when wearing [your/his/her] glasses]? Would you say… [*Read response categories*]

1. No difficulty
2. Some difficulty
3. A lot of difficulty
4. Cannot do at all
7. Refused
9. Don't know

[*Note: This item is Question 1 in the WG Short Set on Functioning.*]

<u>OPTIONAL</u> *Vision questions VIS_3 and VIS_4 are optional:*

VIS_3 [Do/does] [you/he/she] have difficulty clearly seeing someone's face across a room [*If VIS_1 = 1:* even when wearing [your/his/her] glasses]? Would you say… [*Read response categories*]

1. No difficulty
2. Some difficulty
3. A lot of difficulty
4. Cannot do at all
7. Refused
9. Don't know

VIS_4 [Do/does] [you/he/she] have difficulty clearly seeing the picture on a coin [*If VIS_1 = 1:* even when wearing [your/his/her] glasses]? Would you say… [*Read response categories*]?

1. No difficulty
2. Some difficulty
3. A lot of difficulty
4. Cannot do at all
7. Refused
9. Don't know

[*Note*: *Countries may choose to replace "the picture of a coin" with an equivalent item.]*

HEARING

HEAR_1 [Do/Does] [you/he/she] use a hearing aid?

1. Yes
2. No
7. Refused
9. Don't know

HEAR_2 [Do/Does] [you/he/she] have difficulty hearing, [*If HEAR_1 = 1:* even when using a hearing aid(s)]? Would you say… [*Read response categories*]

1. No difficulty
2. Some difficulty
3. A lot of difficulty
4. Cannot do at all
7. Refused
9. Don't know

[*Note: This item is Question 2 in the WG Short Set on Functioning.*]

<u>OPTIONAL</u> Hearing question HEAR_3 is optional:

HEAR_3 How often [do/does] [you/he/she] use [your/his/her] hearing aid(s)? Would you say… [*Read response categories*]

1. All of the time
2. Some of the time
3. Rarely
4. Never
7. Refused
9. Don't know

HEAR_4 [Do/does] [you/he/she] have difficulty hearing what is said in a conversation with one other person in a quiet room [*If HEAR_1 = 1:* even when using [your/his/her] hearing aid(s)]? Would you say… [*Read response categories*]

1. No difficulty
2. Some difficulty
3. A lot of difficulty
4. Cannot do at all
7. Refused
9. Don't know

HEAR_5 [Do/does] [you/he/she] have difficulty hearing what is said in a conversation with one other person in a noisier room [*If HEAR_1 = 1:* even when using [your/his/her] hearing aid(s)]? Would you say… [*Read response categories*]

1. No difficulty
2. Some difficulty
3. A lot of difficulty
4. Cannot do at all
7. Refused
9. Don't know

MOBILITY

MOB_1 [Do/Does] [you/he/she] have difficulty walking or climbing steps? Would you say… [*Read response categories*]

1. No difficulty
2. Some difficulty
3. A lot of difficulty
4. Cannot do at all
7. Refused
9. Don't know

[*Note: This item is Question 3 in the WG Short Set on Functioning.*]

MOB_2 [Do/does] [you/he/she] use any equipment or receive help for getting around?

1. Yes
2. No (*Skip to MOB_4.*)
7. Refused (*Skip to MOB_4.*)
9. Don't know (*Skip to MOB_4.*)

MOB_3 [Do/does] [you/he/she] use any of the following?

Interviewer: Read the following list and record all affirmative responses:

		1. Yes	2. No	*7. Refused*	*9. Don't Know*
A.	Cane or walking stick?				
B.	Walker or Zimmer frame?				
C.	Crutches?				
D.	Wheelchair or scooter?				
E.	Artificial limb (leg/foot)?				
F.	Someone's assistance?				
G.	Other (please specify):				

MOB_4 [Do/Does] [you/he/she] have difficulty walking 100 meters on level ground, that would be about the length of one football field or one city block [*If MOB_2 = 1:* without the use of [your/his/her] aid]? Would you say… [*Read response categories*]

1. No difficulty
2. Some difficulty
3. A lot of difficulty
4. Cannot do at all *(Skip to MOB_6.)*
7. Refused
9. Don't know

[*Note: Allow national equivalents for 100 metres.*]

MOB_5 [Do/Does] [you/he/she] have difficulty walking half a km on level ground, that would be the length of five football fields or five city blocks [*If MOB_2 = 1:* without the use of [your/his/her] aid]? Would you say… [*Read response categories*]

1. No difficulty
2. Some difficulty
3. A lot of difficulty
4. Cannot do at all
7. Refused
9. Don't know

[*Note: Allow national equivalents for 500 metres.*]

MOB_6 [Do/Does] [you/he/she] have difficulty walking up or down 12 steps? Would you say… [*Read response categories*]

1. No difficulty
2. Some difficulty
3. A lot of difficulty
4. Cannot do at all
7. Refused
9. Don't know

If MOB_2 = 2, skip to next section.
If MOB_3 = D "Wheelchair or scooter", skip to next section.

MOB_7 [Do/Does] [you/he/she] have difficulty walking 100 meters on level ground, that would be about the length of one football field or one city block, when using [your/his/her] aid? Would you say… [*Read response categories*]

1. No difficulty
2. Some difficulty
3. A lot of difficulty
4. Cannot do at all *(Skip MOB_8.)*
7. Refused
9. Don't know

MOB_8 [Do/Does] [you/he/she] have difficulty walking half a km on level ground, that would be the length of five football fields or five city blocks, when using [your/his/her] aid? Would you say… [*Read response categories*]

1. No difficulty
2. Some difficulty
3. A lot of difficulty
4. Cannot do at all
7. Refused
9. Don't know

COMMUNICATION

COM_1 Using [your/his/her] usual language, [do/does] [you/he/she] have difficulty communicating, for example understanding or being understood? Would you say… [*Read response categories*]

1. No difficulty
2. Some difficulty
3. A lot of difficulty
4. Cannot do at all
7. Refused
9. Don't know

[*Note: This item is Question 6 in the WG Short Set on Functioning.*]

COM_2 [Do/does] [you/he/she] use sign language?

1. Yes
2. No
7. Refused
9. Don't know

COGNITION (REMEMBERING)

COG_1 [Do/does] [you/he/she] have difficulty remembering or concentrating? Would you say… [*Read response categories*]

1. No difficulty
2. Some difficulty
3. A lot of difficulty
4. Cannot do at all
7. Refused
9. Don't know

[*Note: This item is Question 4 in the WG Short Set on Functioning.*]

COG_2 [Do/does] [you/he/she] have difficulty remembering, concentrating, or both? Would you say… [*Read response categories*]

1. Difficulty remembering only
2. Difficulty concentrating only (S*kip to next section.)*
3. Difficulty with both remembering and concentrating
7. Refused
9. Don't know

COG_3 How often [do/does] [you/he/she] have difficulty remembering? Would you say… [*Read response categories*]

1. Sometimes
2. Often
3. All of the time
7. Refused
9. Don't know

COG_4 [Do/does] [you/he/she] have difficulty remembering a few things, a lot of things, or almost everything? Would you say… [*Read response categories*]

1. A few things
2. A lot of things
3. Almost everything
7. Refused
9. Don't know

SELF-CARE

SC_SS [Do/does] [you/he/she] have difficulty with self care, such as washing all over or dressing? Would you say… [*Read response categories*]

1. No difficulty
2. Some difficulty
3. A lot of difficulty
4. Cannot do at all
7. Refused
9. Don't know

[*Note: This item is Question 5 in the WG Short Set on Functioning.*]

UPPER BODY

UB_1 [Do/Does] [you/he/she] have difficulty raising a 2 liter bottle of water or soda from waist to eye level? Would you say… [*Read response categories*]

1. No difficulty
2. Some difficulty
3. A lot of difficulty
4. Cannot do at all
7. Refused
9. Don't know

UB_2 [Do/Does] [you/he/she] have difficulty using [your/his/her] hands and fingers, such as picking up small objects, for example, a button or pencil, or opening or closing containers or bottles? Would you say… [*Read response categories*]

1. No difficulty
2. Some difficulty
3. A lot of difficulty
4. Cannot do at all
7. Refused
9. Don't know

AFFECT (ANXIETY AND DEPRESSION)

Proxy respondents may be omitted from this section, at country's discretion.

Interviewer: If respondent asks whether they are to answer about their emotional states after taking mood-regulating medications, say: "Please answer according to whatever medication [you were/he was/she was] taking."

ANX_1 How often [do/does] [you/he/she] feel worried, nervous or anxious? Would you say… [*Read response categories*]

1. Daily
2. Weekly
3. Monthly
4. A few times a year
5. Never

7. Refused
9. Don't know

ANX_2 [Do/Does] [you/he/she] take medication for these feelings?

1. Yes
2. No (*If "Never" to ANX_1 and "No" to ANX_2, skip to DEP_1.)*

7. Refused
9. Don't know

ANX_3 Thinking about the last time [you/he/she] felt worried, nervous or anxious, how would [you/he/she] describe the level of these feelings? Would [you/he/she] say… [*Read response categories*]

1. A little
2. A lot
3. Somewhere in between a little and a lot

7. Refused
9. Don't know

DEP_1 How often [do/does] [you/he/she] feel depressed? Would [you/he/she] say… [*Read response categories*]

1. Daily
2. Weekly
3. Monthly
4. A few times a year
5. Never

7. Refused
9. Don't know

DEP_2 [Do/Does] [you/he/she] take medication for depression?

1. Yes
2. No (*If "Never" to DEP_1 and "No" to DEP_2, skip to next section.)*
7. Refused
9. Don't know

DEP_3 Thinking about the last time [you/he/she] felt depressed, how depressed did [you/he/she] feel? Would you say… [*Read response categories*]

1. A little
2. A lot
3. Somewhere in between a little and a lot
7. Refused
9. Don't know

PAIN

Proxy respondents may be omitted from this section, at country's discretion.

Interviewer: If respondent asks whether they are to answer about their pain when taking their medications, say: "Please answer according to whatever medication [you were/he was/she was] taking."

PAIN_1 In the past 3 months, how often did [you/he/she] have pain? Would you say… [*Read response categories*]

1. Never (*If "Never" to PAIN_1, skip to next section.)*
2. Some days
3. Most days
4. Every day
7. Refused
9. Don't know

PAIN_2 Thinking about the last time [you/he/she] had pain, how much pain did [you/he/she] have? Would you say… [*Read response categories*]

1. A little
2. A lot
3. Somewhere in between a little and a lot
7. Refused
9. Don't know

FATIGUE

Proxy respondents may be omitted from this section, at country's discretion.

TIRED_1 In the past 3 months, how often did [you/he/she] feel very tired or exhausted? Would you say… [*Read response categories*]

1. Never (*If "Never" to TIRED_1, skip to next section.)*
2. Some days
3. Most days
4. Every day
7. Refused
9. Don't know

TIRED_2 Thinking about the last time [you/he/she] felt very tired or exhausted, how long did it last? Would you say… [*Read response categories*]

1. Some of the day
2. Most of the day
3. All of the day
7. Refused
9. Don't know

TIRED_3 Thinking about the last time [you/he/she] felt this way, how would you describe the level of tiredness? Would you say… [*Read response categories*]

1. A little
2. A lot
3. Somewhere in between a little and a lot
7. Refused
9. Don't know
